# Association between High-Sensitivity C-Reactive Protein and N-Terminal Pro-B-Type Natriuretic Peptide in Patients with Hepatitis C Virus Infection

**DOI:** 10.1155/2012/730923

**Published:** 2012-05-08

**Authors:** Wenliang Che, Buchun Zhang, Wenling Liu, Yidong Wei, Yawei Xu, Dayi Hu

**Affiliations:** ^1^Department of Cardiology, Shanghai Tenth People's Hospital, Tongji University School of Medicine, Shanghai 200072, China; ^2^Heart Center, Peking University People's Hospital, Beijing 100044, China

## Abstract

*Background.* Prior study showed HCV-infected patients have increased serum N-Terminal Pro-B-Type Natriuretic Peptide (NT-proBNP) and a possible left ventricular diastolic dysfunction. The objectives of the present paper were to investigate the characteristics of hs-CRP and its correlation with clinical profiles including NT-proBNP and echocardiographic variables in HCV-infected patients. *Methods and Results*. A total of 106 HCV-infected patients and 106 control healthy individuals were enrolled. The level of serum hs-CRP (median 1.023 mg/L, range 0.03**∼**5.379 mg/L) was significantly lower in all 106 patients than that in controls (median 3.147 mg/L, range 0.08*~*7.36 mg/L, *P* = 0.012). Although hs-CRP did not correlate significantly with NT-proBNP when all patients and controls were included (*r* = 0.169, *P* = 0.121), simple regression analysis demonstrated a statistically significant linear correlation between hs-CRP and NT-proBNP in HCV-infected patients group (*r* = 0.392, *P* = 0.017). Independent correlates of hs-CRP levels (*R* ^2^ = 0.13) were older age (*β*′ = 0.031, *P* = 0.025) and NT proBNP (*β*′ = 0.024, *P* = 0.017). *Conclusions*. Although the level of serum hs-CRP decreased significantly, there was a significant association between hs-CRP and NT-proBNP in HCV-infected patients.

## 1. Introduction

In the past decade, association of hepatitis C virus (HCV) infection and cardiovascular diseases (CVD) has gained importance because the high occurrence of hepatitis C is a common source of infection worldwide. There have been a series of studies focusing on associations between HCV infection and CVD to investigate whether HCV is a risk factor for development of CVD. Some investigators have reported positive associations between HCV infection and CVD including dilated cardiomyopathy, hypertrophy cardiomyopathy, myocarditis, and coronary artery disease, respectively [1–3]. Our previous study has shown that patients with Hepatitis C virus infection have increased serum N-Terminal Pro-B-Type Natriuretic Peptide (NT-proBNP) and a possible subclinical left ventricular diastolic dysfunctional evidence for a pathogenic link between HCV and CVD [4].

C-reactive protein (CRP) is a biomarker for inflammation and is associated with increased coronary artery disease and cardiovascular events [5, 6]. Regardless of whether CRP plays a causal role in atherothrombosis, this biomarker has been proposed to be useful for improving CVD-risk prediction. In the most recent comprehensive meta-analysis, high-sensitivity C-reactive protein (hs-CRP) was consistently found to be an independent predictor of CVD [7]. Increased hs-CRP has been associated with increased risk for development of hypertension, transient ischemic attack, stroke, peripheral arterial disease, and sudden coronary death [7]. However, the features of hs-CRP in HCV-infected patients and whether hs-CRP is an appropriate surrogate cardiovascular risk marker in HCV-infected population have not been fully addressed.

The aim of this study was to investigate the characteristics of hs-CRP and its correlation with clinical profiles including NT-proBNP and echocardiographic variables in patients with HCV infection.

## 2. Materials and Methods

The present study has been approved by the Ethics Committee of Shanghai Tenth People's Hospital, Tongji University, and performed according to the World Medical Association Declaration of Helsinki.

### 2.1. Study Population

Patient population: from January 2008 to February 2011, one hundred and six consecutive Chinese patients admitted to two different hospitals with well-defined HCV infection were enrolled according to following criteria. Inclusion criteria: positive for serum anti-HCV antibody and HCV RNA. Exclusion criteria: HCV-infected patients treated with interferon *α* in the past six months or less, liver cirrhosis, other types of hepatitis or liver disease of different etiology, other infectious diseases, overt cardiovascular disease (based on documented history and ECG examination), endocrine diseases, lung disease, renal dysfunction (serum creatinine level >2.0 mg/dL), presence or history of neoplastic diseases, pregnant or postpartum women, and present or past history of alcohol or drug abuse.

Control population: healthy individuals whose age and gender matched with HCV-infected patients were selected randomly from the general population and served as a control population. This control group was extracted from a larger sample of 11800 subjects in a population-based survey of plasma cholesterol levels. All enrolled had no documented history of chronic systemic diseases including CVD, renal disorders, liver disorders, and presence or history of neoplastic diseases and autoimmune diseases.

All subjects gave their informed consent for participation in the study, which was approved by the Ethics Committee of Shanghai Tenth People's Hospital, Tongji University.

### 2.2. Laboratory Investigations

Before current initial treatment of HCV infection with interferon *α*, venous blood samples were collected under standardized conditions after an overnight fast and centrifuged at 3,000 g, and then the separated serum was stored at −80°C until analysis. Concentrations of serum hs-CRP were measured using an enzyme-linked immunosorbent assay (ELISA) kits (RapidBio, West Hills, CA, USA). Concentrations of serum NT-proBNP were measured on an Elecsys 2010 instrument (Elecsys 2010, Roche Diagnostics, Mannheim, Germany). Anti-HCV antibody was measured by EIAgen HCV Ab Kit (Adaltis Italia S.p.A). HCV RNA was quantified by Cobas Amplicor HCV Test, Version 2.0 (Roche Molecular Systems, Inc., Pleasanton, CA). Glomerular filtration rate was estimated using the four variable MDRD formula as follows: GFR = 186.3 × (serum creatinine − 1.154) × (age − 0.203) × 1.212 (if black) × 0.742 (if female).

### 2.3. Echocardiographic Measurements

All HCV-infected patients and controls were consecutively submitted to transthoracic echocardiographic evaluation before current initial treatment with interferon *α*. Doppler echocardiographic evaluation was conducted using Philips Sonos 5500, with a 2 ~ 4 MHz transducer and for each evaluated parameter, and three measurements were taken. Two observers made the all assessments, respectively. The following measurable parameters were analyzed: left ventricular diastolic diameter (LVDd), left ventricular posterior wall diastolic thickness (LVPWd), interventricular septum diastolic thickness (IVSd), left ventricular ejection fraction (LVEF), fractional shortening (FS), peak early diastolic transmitral flow (E), peak late diastolic transmitral flow (A), the E/A ratio, and E-wave deceleration time (DT). The early and late diastolic velocities at the septal and lateral mitral annulus were obtained from the apical 4-chamber view using Doppler tissue imaging, and the values were averaged, respectively (E′ and A′), and the ratio of E/E′ was calculated.

### 2.4. Statistical Analysis

Continuous data are expressed as means ± standard deviation for the Gaussian-distributed data and median (range, 25% ~ 75%) for the non-Gaussian-distributed data. Categorical data are expressed as proportions (%). Student's *t*-test or Mann-Whitney *U*-tests was used to determine differences between groups for Gaussian-distributed data or non-Gaussian-distributed data, where appropriate. A chi-square test was used to compare proportions. Linear regression analysis was performed to evaluate the relationship between hs-CRP and clinical profiles.

A *P* value of less than 0.05 was considered statistically significant. Statistical analysis was performed using the statistical package SPSS12.0.1 (SPSS Inc, Chicago, IL).

## 3. Results

### 3.1. Characteristics of the Study Group

A total of 106 patients with HCV infection (38 females, 68 males) aged 24 to 74 (51.1 ± 10.6) years were enrolled and 106 gender-matched controls aged 25 to 73 (50.9 ± 11.0) years were also recruited. The baseline characteristics are presented in Table 1. Height, weight, systolic blood pressure (SBP), diastolic blood pressure (DBP), triglyceride (TG), BMI, and proportion of smokers between groups showed no significant differences. Total cholesterol (TC) level was higher in HCV-infected group (3.81 ± 0.52 mmol/L versus 3.62 ± 0.50 mmol/L, *P* = 0.007). The clinical characteristics of patient group are given in Table 2.

### 3.2. Comparison of Serum hs-CRP and NT-ProBNP Levels between Groups

The level of serum hs-CRP (median 1.023 mg/L, range 0.03 ~ 5.379 mg/L) was significantly lower in all 106 patients than that in controls (median 3.147 mg/L, range 0.08 ~ 7.36 mg/L, *P* = 0.012). The level of serum NT-proBNP (median 64.56 pg/mL, range 21.35 ~ 145.51 pg/mL) in all 106 patients was higher than that in controls (median 16.74 pg/mL, range 12.43 ~ 61.25 pg/mL, *P* < 0.001). The subjects were subdivided into low- and average-to-high risk groups according to hs-CRP cutoff level. Proportion of who had higher hs-CRP levels (≥3 mg/L) was lower in patient group (29, 27.36%; 51, 48.11%; *P* = 0.003). With a cutoff point of 125 pg/mL (the single cutoff point for outpatients under 75 years of age), 17 HCV-infected patients (16.04%) and 4 controls (3.77%) had high NT-proBNP levels (*P* = 0.006). E, E/A, E′, and A′ were significantly lower among patients with high NT-proBNP levels (>125 pg/mL), whereas A, E/E′, and DT were higher (Table 3).

### 3.3. Correlation of Serum hs-CRP Levels and NT-ProBNP Levels

When all patients and controls were included, hs-CRP did not correlate significantly with NT-proBNP (*r* = 0.169, *P* = 0.121). However, in HCV-infected patients group, simple regression analysis demonstrated a statistically significant linear correlation between hs-CRP and NT-proBNP (*r* = 0.392, *P* = 0.017). Furthermore, among those who had higher hs-CRP levels (≥3 mg/L), NT-proBNP was significantly increased (65.41 ~ 157.63 pg/mL versus 22.56 ~ 42.41 pg/mL, *P* < 0.001). In addition, these patients had elder age (59.37 ± 10.12 versus 51.97 ± 11.81, *P* = 0.002) and higher BMI (27.21 ± 3.22 versus 22.31 ± 3.45, *P* < 0.001).

### 3.4. Correlation of Serum hs-CRP Levels and Echocardiographic Variables in HCV-Infected Patients

Our previous study which enrolled 90 HCV-infected patients has demonstrated that mild diastolic dysfunction maybe exist in HCV-infected patients [4]. At the present study, E, E′, and E/A were lower in HCV-infected patients than that in control group, respectively, and E/E′ was higher (Table 4). Correlation of serum hs-CRP levels and echocardiographic variables were analyzed in patients group. Univariate echocardiographic correlates of serum hs-CRP levels are presented in Table 5. Simple regression analysis demonstrated a statistically significant linear correlation between hs-CRP levels and mitral E/E′  (*r* = 0.114, *P* = 0.024).

### 3.5. Independent Correlates of Serum hs-CRP Levels

The serum hs-CRP levels between sexes showed no significant differences. There was no significant correlation between level of hs-CRP and serum HCV RNA titer (*r* = −0.197, *P* = 0.242). Age and BMI were positively correlated with Log hs-CRP, respectively (*r* = 0.332, *P* < 0.001; *r* = 0.124, *P* = 0.021). Entering baseline characteristics and echocardiographic that were significant on univariate analysis into the multivariate analysis revealed that the significant independent correlates were older age (*β*′ = 0.031, *P* = 0.025), NT-proBNP (*β*′ = 0.024, *P* = 0.017), and *R* ^2^ = 0.13  (*P* = 0.016) for the multivariate model (Table 6).

## 4. Discussion

Our study is the first to investigate association between serum hs-CRP and NT-proBNP levels in HCV-infected patients. The most important result is that hs-CRP levels were associated with NT-proBNP levels in HCV-infected patients, although serum hs-CRP level decreased significantly in HCV-infected patients.

CRP is a component of the “acute phase response” associated with infection, inflammation, and tissue damage. Over the last decades, evidence has accumulated that systemic inflammatory activity plays a key pathogenic role in atherosclerosis and CVD. Elevated serum CRP level, as detected by the hs-CRP assay, has been shown to be a stronger predictor of incident cardiovascular events in healthy men than LDL cholesterol and to be additive to the Framingham risk score [8] and was consistently found to be an independent predictor of CVD [7]. Interestingly, in our study, hs-CRP concentrations were markedly lower in patients group, which appears paradoxical in the presence of chronic inflammation. While this finding has been reported in previous study in 1996 [9], similarly, previous study showed HCV-infected patients initiating interferon alfa-2b treatment had lower baseline CRP than uninfected controls [10]. Floris-Moore et al. reported that an association of lower serum lipid and CRP levels with HCV infection in HIV-infected men [11]. In addition, Skowroński et al. reported that CRP levels in diabetic HCV patients were lower than that in diabetic patients without HCV infection [12]. On the contrary, Huang et al. showed that the mean hs-CRP level of chronic HCV infection patients was significantly higher than that of healthy controls [13]. Since interferon *α* therapy may be associated with the levels of hs-CRP [10, 13], patients treated with interferon *α* from less than six months should be excluded in Huang's study. The exact mechanism for that hepatitis C linked with lower hs-CRP level is not known. The present data showed that hs-CRP did not correlate with serum HCV RNA titer. Thus, it suggests that other factors, rather than viral replication, may contribute to HCV-associated reduction in hs-CRP levels. Impaired liver function associated with chronic hepatitis C may lead to depressed production of hs-CRP.

These paradoxical findings highlight the need to assess the predictive value of hs-CRP as a surrogate cardiovascular risk marker in HCV-infected population. In present study, although correlation between hs-CRP and NT-proBNP was not found when all patients and controls were included, there was a statistically significant linear correlation between NT-proBNP and hs-CRP in HCV-infected patients group. NT-proBNP has been demonstrated to be independent risk markers in heart failure patients [14], and in patients with acute coronary syndrome [15, 16], and also in nonhospitalized subjects without known cardiovascular disease [17]. One study has showed that, in patients with heart failure from HCV myocarditis, NT-proBNP is a more sensitive marker of myocardial injury than cardiac troponins [18]. Antonelli et al. demonstrated that patients with hepatitis C showed significantly higher plasma NT-proBNP levels than healthy controls [19]. Our previous data showed that increased NT-proBNP might be a marker for detecting subclinical LV diastolic dysfunction in HCV-infected patients [4]. The finding of the present study obtained in HCV-infected patients with different serum NT-proBNP levels was in line with the previous result, which showed an evidence for diastolic dysfunction in HCV-infected patients with high NT-proBNP levels (>125 pg/mL). The present data demonstrated that hs-CRP levels possibly associated with cardiovascular events risk in HCV-infected population, although hs-CRP levels of patient group decreased compared with healthy controls. Furthermore, we investigated whether hs-CRP-associated LV diastolic dysfunction. The univariate linear regression analysis demonstrated a significant correlation between hs-CRP and E/E′ in HCV-infected patients. However, this factor was the significant confounding factors, because that significant independent correlates of serum hs-CRP were older age and NT-proBNP for the multivariate model.

Some limitations of our study deserve to be discussed. Firstly, the number of patients was relatively small and probably the characteristics differ from the general population. Secondly, follow-up data that may show elevated hs-CRP levels are associated with adverse cardiac events are lacking.

## 5. Conclusion

In summary, the findings of the present study may have important implications for patients with HCV infection. Although the level of serum hs-CRP decreased significantly, there was a significant association between hs-CRP and NT-proBNP in HCV-infected patients. The predictive value and application of hs-CRP as a surrogate cardiovascular risk marker should have been evaluated in CVD patients in conjunction with HCV infection, and screening for HCV should be considered prior to assessment of cardiovascular risk, because the results may affect the cardiovascular risk scoring.

## Figures and Tables

**Table 1:** Baseline characteristics of subjects.

Variables	Patients (*N* = 106)	Controls (*N* = 106)	*P* value
Height (cm)	168.4 ± 5.3	168.9 ± 6.1	0.391
Weight (kg)	73.5 ± 8.4	72.9 ± 7.7	0.588
SBP (mmHg)	124.6 ± 13.1	125.3 ± 12.3	0.689
DBP (mmHg)	76.3 ± 8.0	75.6 ± 9.1	0.553
TC (mmol/L)*	3.81 ± 0.52	3.62 ± 0.50	**0.007 **
Triglycerides (mmol/L)	1.12 ± 0.46	1.17 ± 0.57	0.483
BMI	22.26 ± 3.34	22.52 ± 3.17	0.562
Smoker (*n*, %)	12, 11.3	14, 13.2	0.832

Abbreviations: SBP, Systolic blood pressure; DBP, Diastolic blood pressure; TC, total cholesterol.

Data are expressed as mean ± SD.

*A *P* value of less than 0.05 was considered statistically significant.

**Table 2:** Clinical characteristics of patients group.

Variables	Patients
Duration of hepatitis C (yr; means ± SD)	5.2 ± 3.7
HCV RNA (copies/mL; median (25% ~ 75%))	3.74 × 10^6^ (2.13 × 10^5^ ~ 2.43 × 10^7^)
Serum albumin (g/L; means ± SD)	37.41 ± 7.14
AST (IU/L; median (25% ~ 75%))	51.6 (28.4 ~ 84.6)
ALT (IU/L; median (25% ~ 75%))	42.4 (26.9 ~ 81.4)
LDH (IU/L; means ± SD)	181.4 ± 46.4
Serum creatinine (mg/dL; median (25% ~ 75%))	0.81 (0.69 ~ 0.93)
eGFR (mL·min^−1^·(1.73 m^−2^)^−1^; means ± SD)	95.6 ± 10.4

Abbreviations: AST, aspartate aminotransferase; ALT, alanine aminotransferase; LDH, lactate dehydrogenase; eGFR, estimated glomerular filtration rate.

**Table 3:** Echocardiographic features of patients with different serum NT-proBNP Levels.

Variables	High level	Normal level	*P* value
NT-proBNP	>125 pg/mL	<125 pg/mL	—
No.	17	89	—
LVDd (mm)	47.62 ± 4.38	45.90 ± 4.68	0.163
LVPWd (mm)	9.24 ± 1.26	9.10 ± 2.05	0.787
IVSd (mm)	9.10 ± 1.64	8.95 ± 1.72	0.741
E (cm/s)*	70.06 ± 17.40	79.49 ± 16.04	**0.031**
A (cm/s)*	98.90 ± 19.72	73.27 ± 12.40	**<0.01**
E/A*	0.75 ± 0.16	1.14 ± 0.36	**<0.01**
DT (ms)*	238.4 ± 52.7	197.4 ± 24.9	**<0.01**
E′ (cm/s)*	6.24 ± 1.24	9.91 ± 2.61	**<0.01**
A′ (cm/s)*	9.71 ± 2.84	11.90 ± 2.94	**0.006 **
E/E^′∗^	12.77 ± 3.15	8.31 ± 1.94	**<0.01**
LVEF (%)	64.90 ± 6.62	67.05 ± 4.01	0.075
FS (%)	39.52 ± 5.32	41.62 ± 4.68	0.100

Abbreviations: LVDd, left ventricular diastolic diameter; LVPWd, left ventricular posterior wall diastolic thickening; IVSd, interventricular septum diastolic thickening; E, peak early diastolic transmitral flow; A, peak late diastolic transmitral flow; E′, peak early diastolic annular velocity; A′, peak late diastolic annular velocity; E/E′, the ratio of E to E′; DT, deceleration time; LVEF, left ventricular ejection fraction; FS, fractional shortening.

Data are expressed as mean ± SD.

*A *P* value of less than 0.05 was considered statistically significant.

**Table 4:** Echocardiographic measurements between groups.

Variables	Patients (*N* = 106)	Controls (*N* = 106)	*P* value
LVDd (mm)	48.23 ± 4.31	47.87 ± 3.54	0.507
LVPWd (mm)	9.46 ± 1.73	9.13 ± 1.82	0.177
IVSd (mm)	9.75 ± 1.66	9.82 ± 1.45	0.744
E (cm/s) *	69.84 ± 13.43	87.54 ± 16.27	**<0.01**
A (cm/s)	62.13 ± 10.37	59.84 ± 11.32	0.126
E/A*	1.13 ± 0.34	1.47 ± 0.41	**<0.01**
DT (ms)	175.4 ± 26.7	177.4 ± 24.9	0.573
E′ (cm/s)*	7.28 ± 2.42	9.83 ± 2.74	**<0.01**
A′ (cm/s)	10.51 ± 2.84	9.98 ± 2.94	0.183
E/E^′∗^	9.77 ± 2.45	8.86 ± 2.74	**0.012**
LVEF (%)	64.73 ± 7.45	66.46 ± 7.73	0.099
FS (%)	38.54 ± 4.62	39.15 ± 4.82	0.348

Abbreviations: LVDd, left ventricular diastolic diameter; LVPWd, left ventricular posterior wall diastolic thickening; IVSd, interventricular septum diastolic thickening; E, peak early diastolic transmitral flow; A, peak late diastolic transmitral flow; E′, peak early diastolicannular velocity; A′, peak late diastolic annular velocity; E/E′, the ratio of E to E′; DT, deceleration time; LVEF, left ventricular ejection fraction; FS, fractional shortening

Data are expressed as mean ± SD.

*A P value of less than 0.05 was considered statistically significant.

**Table 5:** Correlation coefficients of linear regression analysis between hs-CRP and echocardiographic parameters in patients group.

	Log hs-CRP	
Variable	*r* Value	*P* Value
LVDd (mm)	0.134	0.114
LVPWd (mm)	0.024	0.387
IVSd (mm)	0.162	0.643
LVEF (%)	−0.250	0.521
FS (%)	−0.630	0.324
E (cm/s)	0.377	0.721
A (cm/s)	0.245	0.153
E/A	0.312	0.330
DT (ms)	−0.162	0.176
E′ (cm/s)	0.234	0.087
A′ (cm/s)	0.456	0.063
E/E^′∗^	0.114	**0.024**

Abbreviations: LVDd, left ventricular diastolic diameter; LVPWd, left ventricular posterior wall diastolic thickening; IVSd, interventricular septum diastolic thickening; LVEF, left ventricular ejection fraction; FS, fractional shortening; E, peak early diastolic transmitral flow; A, peak late diastolic transmitral flow; DT, deceleration time; E, peak early diastolic annular velocity; A′, peak late diastolic annular velocity; E/E′, the ratio of E to E′

*A *P* value of less than 0.05 was considered statistically significant.

**Table 6:** Multivariable linear regression for Log hs-CRP as dependent variable.

Variable	*β*	*β*′	*P* value
age (y)*	0.044	0.031	**0.025**
NT-proBNP*	0.012	0.024	**0.017**
BMI	0.027	0.164	0.098
E/E′	0.124	0.235	0.138

Abbreviations: E/E′, the ratio of E to E′; *β*: Regression coefficient; *β*′: standardized coefficient

*A *P* value of less than 0.05 was considered statistically significant.
